# Ultra-high resolution profiles of macular intra-retinal layer thicknesses and associations with visual field defects in primary open angle glaucoma

**DOI:** 10.1038/srep41100

**Published:** 2017-02-07

**Authors:** Qi Chen, Shenghai Huang, Qingkai Ma, Huiling Lin, Mengmeng Pan, Xinting Liu, Fan Lu, Meixiao Shen

**Affiliations:** 1 School of Ophthalmology and Optometry, Wenzhou Medical University, Wenzhou, Zhejiang, China

## Abstract

The structural characteristics of the outer retinal layers in primary open angle glaucoma (POAG) are still controversial, and these changes, along with those in the inner retinal layers, could have clinical and/or pathophysiological significance. A custom-built ultra-high resolution optical coherence tomography (UHR-OCT) combined with an automated segmentation algorithm can image and measure the eight intra-retinal layers. The purpose of this study is to determine the thickness characteristics of the macular intra-retinal layers, especially the outer layers, in POAG patients. Thirty-four POAG patients (56 eyes) and 33 normal subjects (63 eyes) were enrolled. Thickness profiles of the eight intra-retinal layers along a 6-mm length centred on the fovea at the horizontal and vertical meridians were obtained and the regional thicknesses were compared between two groups. The associations between the thicknesses of each intra-retinal layer and the macular visual field (VF) sensitivity were then analysed. POAG affected not only the inner retinal layers but also the photoreceptor layers and retinal pigment epithelium of the outer retina. However, the VF loss was correlated mainly with the damage of the inner retinal layers. UHR-OCT with automated algorithm is a useful tool in detecting microstructural changes of macula with respect to the progression of glaucoma.

To promote better monitoring, intervention, and therapy for glaucoma and other severely blinding diseases, it is important to understand the retinal remodeling that is associated with optic neuropathies. The predominant damage to the retina in primary open angle glaucoma (POAG) is a progressive optic neuropathy characterized by the loss of the retinal ganglion cells and associated axons and dendrites^1^. It is widely accepted that the thicknesses of the inner retinal layers, including the retinal nerve fibre layer (NFL), ganglion cell layer (GCL), and the inner plexiform layer (IPL) are significantly decreased in POAG patients^2^,^3^. These changes are also associated with visual field (VF) defects^4^,^5^.

Some functional studies have demonstrated that the outer retinal layers, which include the photoreceptor and the retinal pigment epithelium (RPE) layers, are also dysfunctional in POAG patients. Vincent *et al*.^6^, using electroretinography, reported that patients with advanced glaucoma had noticeable regional outer retinal dysfunction in the central ±24^o^. Pokorny *et al*.^7^ considered that the glaucomatous patients often present with blue-yellow color defects, implying that the neuropathy might include disruptions of the outer retinal layers. However, the characteristics of the outer retinal layers in POAG are still controversial^6^,^8^,^9^,^10^,^11^,^12^,^13^.

To explore the mechanism of POAG and to allow more precise monitoring of disease progression, it is necessary to image and segment more retinal structures, even down to the cellular level. Ultra-high resolution optical coherence tomography (UHR-OCT), based on spectral-domain principles, has an axial resolution of ~3 μm, which enables detailed detection of intra-retinal structures that were previously only available by histopathology^14^,^15^. In our previous study, we used a custom-built UHR-OCT instrument combined with a custom-developed automated segmentation algorithm to measure the thicknesses of eight intra-retinal layers in 6-mm scans centred on the fovea. The instrumentation and associated software had superior repeatability and reproducibility in the healthy eyes^16^, and has been successfully applied to high myopia patients^17^.

Because potential photoreceptor and RPE damage in POAG may have clinical or pathophysiological significance, in the present study we sought to determine by UHR-OCT the thickness characteristics of the macular intra-retinal layers, especially the outer layers in POAG patients. We also analysed the associations between the thicknesses of each intra-retinal layer of both the inner and outer retina and VF defects.

## Results

A total of 34 patients (56 eyes) clinically diagnosed with POAG and 33 age- and sex-matched control subjects (63 eyes) were recruited for this study. Their demographic properties were listed in Table 1.

There were no significant differences between the two repeated measurements of intra-retinal layer thickness as calculated by the automated algorithm based on UHR-OCT images (paired t-tests, all p-values > 0.05, Table 2). The standard deviation (SD) of the differences of the two measurements ranged from 0.534 to 2.181 μm (Table 2). The intraclass correlation coefficients (ICCs) for the NFL, GCL + IPL, inner nuclear layer (INL), Henle fibre layer + outer nuclear layer (HFL + ONL), outer segments (OS) of the photoreceptor, interdigitation zone (IZ) + RPE (IZ + RPE), and total layer thicknesses were between 0.976 and 0.999 in the horizontal meridian and between 0.968 and 0.998 in the vertical meridian (Table 2). For the outer plexiform layer (OPL) and the myoid and ellipsoid zone (MEZ), the ICCs were 0.820 and 0.916 in the horizontal meridian and 0.777 and 0.883 in the vertical meridian, respectively. The coefficients of repeatability (CORs) for the layer thicknesses in the horizontal meridian ranged from 0.26% to 5.21%, except for the OPL, which was 6.49% (Table 2). The CORs in the vertical meridian were between 0.33% and 5.85%, except for the NFL, which was 8.11%, and the OPL, which was 7.64%.

**Figure 1:**
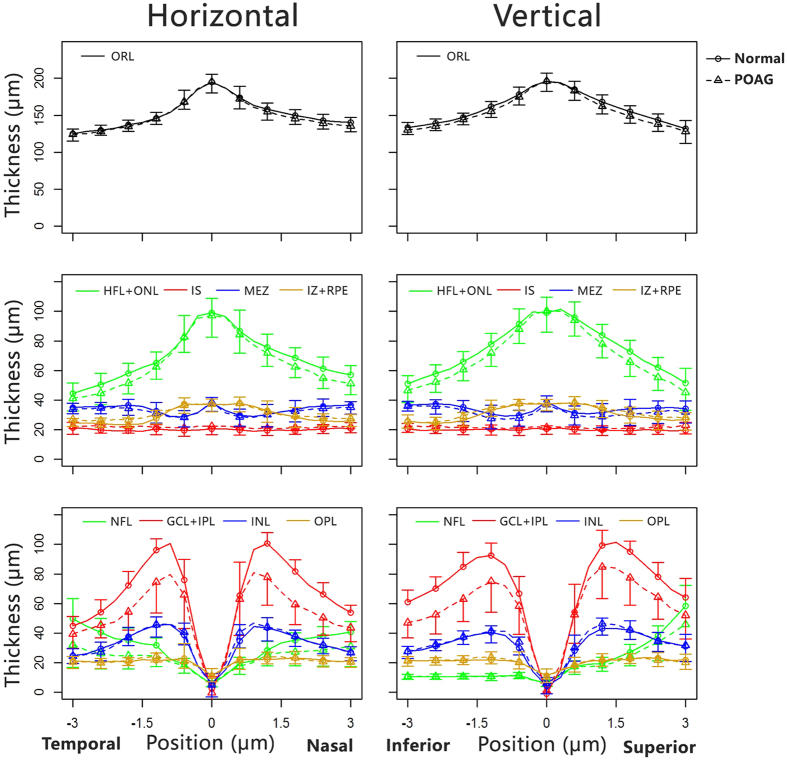
Comparison of thickness profiles for eight intra-retinal layers determined by automated segmentation of UHR-OCT images of normal and POAG eyes. Thickness profiles of the ORL, the intra-retinal layers (OPL, HFL + ONL, MEZ, OS and IZ + RPE) of the outer retina, and the intra-retinal layers (NFL, GCL + IPL, INL) of the inner retina. Images along the horizontal (left) and vertical (right) meridians were averaged over 56 POAG eyes (dotted lines) and 63 normal healthy eyes (solid lines). Error bars represent standard error of the mean. ORL: outer retinal layer; NFL: nerve fibre layer; GCL + IPL: ganglion cell layer and inner plexiform layer; INL: inner nuclear layer; OPL: outer plexiform layer; HFL + ONL: Henle fibre layer and outer nuclear layer; MEZ: myoid and ellipsoid zone; OS: outer segment of photoreceptors; IZ + RPE: interdigitation zone and retinal pigment epithelium/Bruch complex.

**Figure 2:**
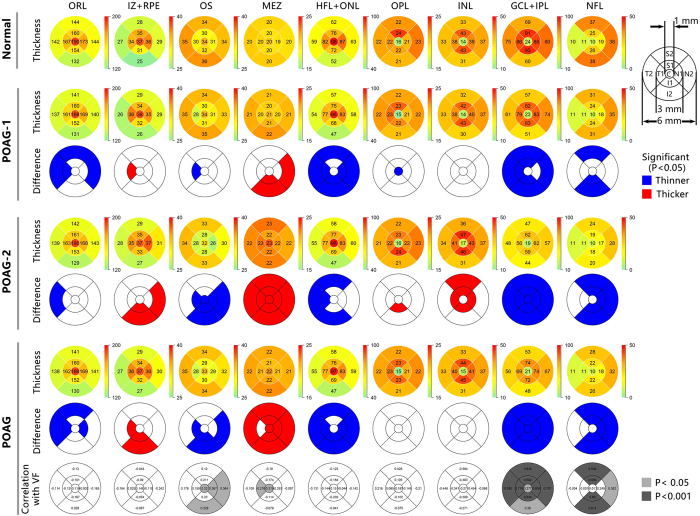
Thickness maps of the outer retinal layer and eight intra-retinal layers in nine sectors for normal and POAG subjects. Thickness maps: Row 1, normal group; Row 2: mild POAG group (POAG-1); Row 4: moderate to advanced POAG group (POAG-2); Row 6: total POAG group. The thickness for each of the nine macular regions is indicated by the color bar to the right of each map. Thickness difference maps between the normal group and the POAG groups: Row 3, mild POAG group (POAG-1); Row 5: moderate to advanced POAG group (POAG-2); Row 7: total POAG group. Blue indicates significantly thinner layers (P < 0.05) and red indicates significantly thicker layers (P < 0.05) compared to the normal group. Correlation map: Row 8, correlation between the thickness and the macular visual field (VF) sensitivity (dB) in all regions for total POAG group. C, central region; S1, superior region in the internal ring; S2, superior region in the external ring; I1, inferior region in the inner ring; I2, inferior region in the outer ring; N1, nasal region in the internal ring; N2, nasal region in the external ring; T1, temporal region in the internal ring; T2, temporal region in the external ring. ORL: outer retinal layer; NFL: nerve fibre layer; GCL + IPL: ganglion cell layer and inner plexiform layer; INL: inner nuclear layer; OPL: outer plexiform layer; HFL + ONL: Henle fibre layer and outer nuclear layer; MEZ: myoid and ellipsoid zone; OS: outer segment of photoreceptors; IZ + RPE: interdigitation zone and retinal pigment epithelium/Bruch complex.

Thickness profiles of the eight intra-retinal layers and the total retina along the 6-mm length centred on the fovea at both the horizontal and vertical meridians were obtained for both the normal and POAG groups (Fig. 1). The thicknesses were analysed in nine regions along the vertical and horizontal meridians (Fig. 2): central fovea (C), superior (S) 1, inferior (I) 1, nasal (N) 1, temporal (T) 1, S2, I2, N2, and T2. The outer retinal layer was thinner in the POAG group in four regions (T1, T2, N1, and S2; P < 0.05 each; Fig. 2). Within the outer retinal layer of the POAG group, the HFL + ONL in almost all the regions (except for the C and S1 regions) and OS in four regions (T1, N2, I1 and I2) were thinner than those of the normal group (P < 0.05). However, the thickness of the MEZ was slightly, but significantly, increased in all regions (P < 0.05) except T1. In addition, the IZ + RPE of POAG eyes were thicker in three regions, T1, I1, and I2 (P < 0.05, Fig. 2). In other regions there was a tendency for it to be thicker though the differences were not significant (P > 0.05). There were no differences in OPL thickness between the two groups in any region (P > 0.05, Fig. 2). For the inner retinal layers, except for the C, T1, and T2 regions, the NFL in POAG eyes was significantly decreased compared to the normal eyes (P < 0.05, Fig. 2). All of the GCL + IPL regions were also thinner in POAG eyes (P < 0.05). The thickness of the INL were similar for the two groups (P > 0.05).

In addition, we divided the 56 POAG eyes into two sub-groups according to the Hodapp-Parrish-Anderson criteria^18^: those with mild POAG (32 eyes, VF mean deviation ≥ −6 dB) and those with moderate to advanced POAG (24 eyes, VF mean deviation < −6 dB to −20 dB). In the preliminary analysis of the sub-groups, we found that the intra-retinal layers were significantly altered, even in the patients with mild POAG (Fig. 2). The changes included decreases in the NFL (S2, I1, and I2), GCL + IPL (all the regions except for C and N1), OPL (C), HFL + ONL (all regions except for C and S1), and OS (T1), and there were increases in the MEZ (N1, I1 and I2) and IZ + RPE (T1) (P < 0.05 for all comparisons). Moreover, the changes of almost all of the above intra-retinal layers were more severe in the moderate to advanced POAG sub-group (P < 0.05, Fig. 2).

In the POAG group, the NFL thickness was significantly correlated with the macular VF sensitivity for regions S1, S2, I1, I2 and N2 (r = 0.362~0.690, P < 0.05, Fig. 2). The thickness of the GCL + IPL in all regions was significantly correlated with the macular VF sensitivity (r = 0.277~0.719, P < 0.05, Fig. 2). For the outer retinal layers, VF sensitivity was negatively correlated with the MEZ in the C and T1 regions (r = −0.319 and −0.276, respectively) and positively correlated with the OS in the C, N1, N2, and I2 regions (r = 0.320~0.367) (P < 0.05 for all, Fig. 2). None of the other intra-retinal layers were correlated with VF sensitivity.

## Discussion

Using an automated segmentation algorithm that detects eight retinal layers in UHR-OCT images, we have confirmed and extended our understanding of the effects of POAG on intra-retinal layer thicknesses and assessed the relationship of those changes to VF changes. Further, through comparison of POAG eyes with age- and sex-matched healthy eyes, we have discovered some new findings in the outer intra-retinal layers of POAG eyes. In the outer retina, the HFL + ONL and OS layers were thinner in POAG eyes. In contrast, the MEZ and RPE were thicker in certain regions. We also found significant thinning of certain inner retinal layers in POAG eyes, i.e., the NFL and GCL + IPL, as previously reported by others^2^,^3^.

Even though several previous functional studies that used electroretinography indicated that the outer retinal layers are damaged in glaucoma^19^,^20^, the morphological changes in this region are still not fully understood and are a topic of debate. Wang *et al*.^21^ reported that the outer retinal layer, including the photoreceptor layers and the OPL, did not change in the glaucoma. However in our study, we found that the outer retinal layer, including the OPL, photoreceptor layer, and IZ + RPE, was slightly but significantly thinner in some regions. These findings are similar to those of Tan *et al*.^22^ who considered that the entire outer retinal layer had minimal thinning (3%). In addition, there is no agreement regarding changes within the photoreceptor layers. Kurtiset *et al*.^10^ found no significant differences between control and POAG eyes in comparisons of photoreceptor density, ONL height, or photoreceptor number. However, Guo *et al*.^11^ found that the ONL was thinner in murine eyes under high intraocular tension, which is consistent with the results of our results reported here and of Lei *et al*.’s study^23^. In addition, Werner *et al*.^12^ and Choi *et al*.^13^, using adaptive optics OCT, also showed a reduction in cone density in patients with glaucoma. However, Fan *et al*.^9^ found that the ONL was thicker in the fovea but not in the parafovea (1.5 mm, S1, I1, N1 and T1), a state possibly arising from the swelling of cone cells and enlargement of the nuclei as mentioned by Nork *et al*.^24^. In the present study, this layer did not change in the fovea, but it was thinner in the para- and perifovea (S2, I2, N2 and T2). In addition, Fan *et al*.^9^ reported that the photoreceptor inner and outer segments were unchanged in POAG. In contrast, Werner *et al*.^12^ and Choi *et al*.^13^ reported that the OS were shorter and the OS layer was thinner in glaucoma eyes, as we also found. The differences among the above studies might be due to differences in measurement methods, differences in ethnicities, and/or differences inpatient profiles, such as age, refractive error, axial length, and/or the severity of glaucoma in each study.

We also found that the IZ + RPE layer was thicker in some regions of POAG eyes. Little is documented about the thickness changes of this layer in the glaucoma. A previous study reported that the choroidal blood flow was abnormal in POAG^25^. It is possible that choroidal dysfunction could affect the IZ + RPE, leading to thickening due to the accumulation of shedding discs^26^. Although the thickness of the IZ + RPE layer increased only in the T1, I1, and I2 regions, other regions also tended to increase. In a previous study, we also found a regional increase in IZ + RPE thickness in eyes with high myopia, which also have abnormal choroid blood flow^17^. The increased thickness is possibly due to the same mechanism in POAG and high myopia eyes.

As previously documented by others^2^,^3^, we also found that the inner retinal layers, including the NFL and the GCL + IPL, were significantly thinner in the POAG group. However, though somewhat thinner than in the control eyes, the differences in thickness of the NFL at the C, T1, and T2 regions were not significant. We hypothesize that because of the natural thinness of the NFL in the temporal and central regions (approximately 10 μm), it is less susceptible to thinning due to glaucoma. How the INL changes in glaucoma is still controversial. Tan *et al*.^22^ and Lei *et al*.^23^ found that the INL was thinner in glaucoma eyes, but Wang *et al*.^21^ reported that there were no significant differences between glaucoma and normal eyes, which is consistent with our study.

In view of the high clinical value of the VF defects in the diagnosis and monitoring of POAG, in the current study we analysed the correlations between the thickness of each intra-retinal layer and the macular VF sensitivity. Even though there were differences between POAG patients and controls in the thickness of several layers of the outer retina, we found that only the MEZ thickness had weak negative correlation with VF sensitivity and that the OS layer thickness had a weak positive correlation with VF sensitivity. This implies that some VF defects in POAG might be due to changes in the outer retinal layers. In addition, we found that the thicknesses of both the NFL and the GCL + IPL were positively correlated with VF sensitivity. This is consistent with the results of Sato *et al*.^4^ and Cho *et al*.^5^ who found significant correlations between the macular VF sensitivity and the GCL + IPL, the ganglion cell complex (comprised of the macular NFL and GCL + IPL), and the peripapillary retinal NFL thicknesses. Therefore, we hypothesize that the VF defects in POAG are due mainly to damage of the inner retinal layers rather than the outer retinal layers.

In this study, we used a UHR-OCT (~3-μm resolution), which resolved the eight intra-retinal layers quite clearly. In addition, we applied an advanced version of the original automated algorithm on the images of POAG eyes after the image magnification was adjusted by the axial length. This adjustment made the analysis much more precise for the eyes with higher refractive error. This automated algorithm successfully segmented high myopia eyes^16^,^17^, but this was the first use on POAG eyes. Therefore, we assessed the SD of the differences as well as the ICC and COR of the two measurements as measures of repeatability. All of the measures were good, ranging from 0.534 to 2.181 μm for SD of differences, from 0.777 to 0.999 for the ICC, and from 0.26% to 8.11% for the COR. Thus this algorithm can be used to automatically measure the thicknesses of the intra-retinal layers in POAG without significant measurement error.

There were limitations in the current study. First, the OCT images were obtained in only two meridians; however, there are probably some local changes outside of these meridians that were not observed. Optimally, such a study would include three-dimensional retinal imaging, which would yield more explicit and interesting results regarding POAG-induced changes in the subtle intra-retinal layers. Second, this was a cross-sectional study, and some of the potentially distinctive changes must be observed through longitudinal study. Third, in the current study, we further divided the 56 POAG eyes into two sub-groups: the mild POAG and the moderate to advanced POAG groups. However, such a grouping was relatively rough only according to the VF mean deviation. Dynamic VF defects as well as the multiple-measure intraocular pressure (IOP) and its fluctuation should be considered for a specific patient, and the relationships between these clinical data and the thickness changes of the intra-retinal layers should be further discussed. These limitations can be resolved in the future by a longitudinal study composed of a larger sample size with more refined groupings according to patient clinical characteristics.

In conclusion, we used automated analysis of UHR-OCT images to assess the thickness profiles of the intra-retinal layers in patients with POAG and in normal control subjects. POAG affected not only the inner retinal layers but also the photoreceptor layers and RPE of the outer retina. The VF loss was correlated mainly with damage of the inner retinal layers. UHR-OCT with automated algorithm is a useful tool in detecting microstructural changes of macula with respect to the progression of glaucoma.

## Methods

### Subjects

This study was approved by the ethics committee of the Eye Hospital of Wenzhou Medical University. The ethics committee approved the screening, inspection, and data collection of these patients, and all patients provided written informed consent. All experiments followed the provisions of the Declaration of Helsinki.

Sixty-seven subjects, including 34 patients diagnosed as POAG and 33 age- and sex-matched control subjects, were enrolled. All subjects had an extensive ophthalmologic examination, including slit-lamp biomicroscopy, subjective refraction, axial length measurement using an optical biometer (IOL Master; Carl Zeiss Meditec, Jena, Germany), IOP measurement by Goldmann applanation tonometry, ophthalmoscopy, and visual field testing (not for controls). Patients with clinical evidence of retinal diseases and/or optic nerve diseases, previous refractive or retinal surgery, opaque refractive media, or hypertension and diabetes were excluded. Normal subjects were presumed to have no VF defects, and they had no structural optic disc abnormalities, no history of IOP > 21 mmHg, and no history of ocular or neurological diseases.

### UHR-OCT and Data Collection

**Figure 3:**
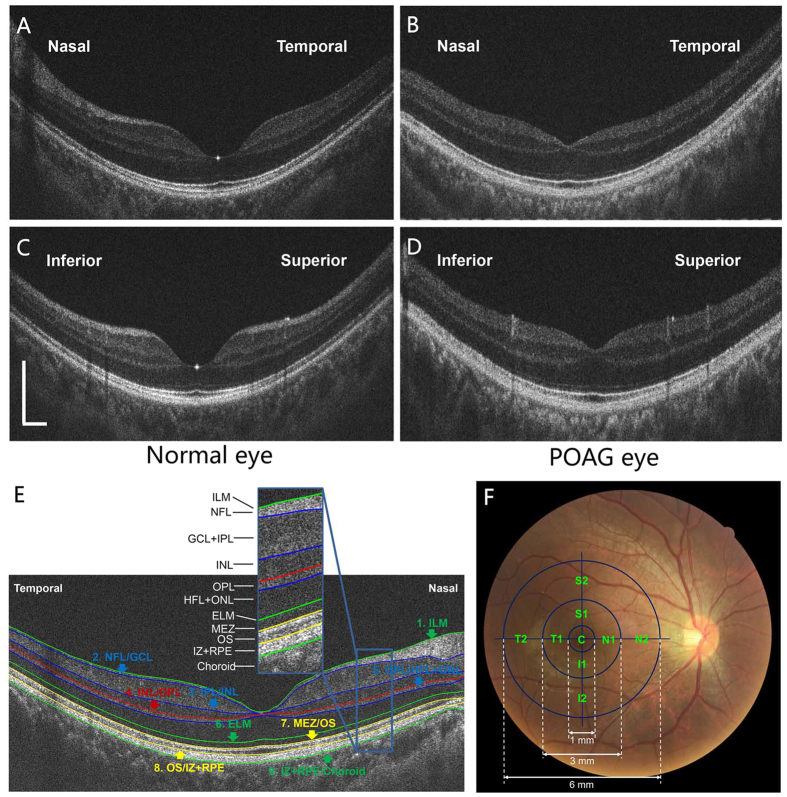
UHR-OCT images of the macula divided into nine regions. (**A**–**D**) UHR-OCT images of a normal eye (left) and a primary open angle glaucoma (POAG) eye (right). Top row, horizontal meridian; Bottom row, vertical meridian. Bars = 500 μm. (**E**) Nine boundaries of the intra-retinal structures from inner to outer retina. The intra-retinal layers were defined as the structures between two neighbouring boundaries: NFL: nerve fibre layer; GCL + IPL: ganglion cell layer and inner plexiform layer; INL: inner nuclear layer; OPL: outer plexiform layer; HFL + ONL: Henle fibre layer and outer nuclear layer; MEZ: myoid and ellipsoid zone; OS: outer segment of photoreceptors; IZ + RPE: interdigitation zone and retinal pigment epithelium/Bruch complex. (**F**) Nine regions divided on the horizontal and vertical meridians around the macular fovea. The inner concentric ring (parafovea) ranged from 1 to 3 mm from the central fovea (**C**) and consisted of the following regions: S1, superior; I1, inferior; N1, nasal; and T1, temporal. The outer concentric ring (perifovea) ranged from 3 to 6 mm from the C and consisted of the following regions: S2, superior; I2, inferior; N2, nasal; and T2, temporal.

A custom-built UHR-OCT with a resolution of approximately ~3 μm in air was adapted onto a slit-lamp system for imaging the posterior segment of the eye, as described previously^16^,^17^. Briefly, the light source was a superluminescent diode (SLD: T840; SuperLum Diodes Ltd., Moscow, Russia) with a center wavelength of 840 nm and a bandwidth of 100 nm. A specially designed spectrometer with a charged-coupled device camera was used to detect the interference spectrum from both sample and reference arms. The power of the incident light to the eye was set to 750 μW, which is well below the safety standard, according to the American National Standards Institute (ANSI Z136.1-2000). The fixation target was computer-controlled and built into the biomicroscope optics. A fundus camera was placed between the dichroic mirror and biomicroscope. An ocular lens (60 diopter; Volk Optical, Mentor, OH, USA) was positioned after the 45° hot mirror. The scan speed of the system was set to 24 K A-scans per second, and the field of the scan was set to approximately 15° to 20°. Cross-line OCT scans (horizontal and vertical scans), each 8 mm in length, were used to acquire the macular retina. Typical retinal UHR-OCT images at the horizontal and vertical meridians of healthy and POAG eyes easily resolved the intra-retinal layers (Fig. 3A–D).

### Image Analysis

To segment the nine boundaries of the intra-retinal layers (Fig. 3E), a dedicated software program based on the gradient information and shortest path search was developed in Matlab (The Mathworks Inc., Natick, MA, USA) for automated image analysis^17^,^27^. Raw macular retinal images of 8 mm in length were acquired using the cross-line scan mode, and the central 6 mm of the fovea was analysed after the image magnification of each eye was adjusted by its axial length. The intra-retinal layer boundaries included the (1) internal limiting membrane, (2) NFL/GCL, (3) IPL/INL, (4) INL/OPL, (5) OPL/HFL + ONL, (6) external limiting membrane, (7) MEZ/OS, (8) OS/IZ + RPE, and the (9) IZ + RPE/choroid.

## Experimental Procedure

### Repeatability of the automated segmentation algorithm applied to POAG eyes

The high repeatability of the developed automated segmentation algorithm in healthy eyes was verified in the previous study^16^. As the characteristics of the intra-retinal layers in the diseased retina may be unique, it was necessary to evaluate the repeatability of the algorithm again before it could be used to analyse the thickness profiles of the glaucomatous retina. Two consecutive images of ten recruited POAG eyes were acquired both at the horizontal and vertical meridians. The eight intra-retinal layers included the NFL, GCL + IPL, INL, OPL, HFL + ONL, MEZ, OS, and IZ + RPE (Fig. 3E). The averaged thickness of each layer in two consecutive images obtained was used to evaluate the repeatability of the automated algorithm.

### Comparisons of the thickness profiles of the eight intra-retinal layers between the normal and glaucoma groups

The thickness profiles for intra-retinal layers along a 6-mm length centred on the fovea in the horizontal and vertical meridians were calculated by subtracting the boundary positions of each of the adjacent layers obtained by automated segmentation along the retinal depth. The horizontal and vertical 6-mm length thickness profiles were then divided into nine regions (Fig. 3F) that were compared between the glaucoma and normal groups. The central fovea was designated as region “C” and included the 1-mm cross line scans of the horizontal and vertical meridians. The thickness of C was determined as the average of the two meridional scans. An inner concentric ring (parafovea) around C was defined to include four regions between 1 and 3 mm from the center of the fovea. Within this concentric ring, the regions were designated as “S1”, “I1”, “N1”, or “T1” representing the superior, inferior, nasal, and temporal regions. An outer concentric ring (perifovea) around C was defined to include four regions between 3 and 6 mm from the center of the fovea. Within this concentric ring, the regions were designated as “S2”, “I2”, “N2”, or “T2” representing the superior, inferior, nasal, and temporal regions.

### Relationship between intra-retinal layer thicknesses and VF defects in POAG eyes

In this study, we analysed the regional relationships between the thickness of each intra-retinal layer in eyes with POAG and the associated VF defects. The statistical methods for testing the correlations between intra-retinal layer thickness and VF defects are describe in the following section.

### Statistical Analysis

All statistical analyses were performed with the Statistical Package for the Social Sciences (SPSS) software (v17.0 for Windows; SPSS Inc., Chicago, IL, USA). Descriptive statistics were determined as means ± standard deviations. Differences between the automated and manual detections of the boundary positions in each A-scan were calculated. After determining that the data for each variable was normally distributed, paired t-tests were used to compare the repeated measures. The COR, expressed as a percent, was calculated as two standard deviations of the differences between measurements divided by the average of each pair of measurements taken on separate visits. The ICC for absolute agreement was calculated to assess the reliability of the repeated measurements. Two independent samples t-tests were used to compare the differences of intra-retinal layer thicknesses in each region between the normal and the glaucoma groups. Correlations of each intra-retinal layer thickness with VF defects in glaucoma were estimated by the Pearson correlation. P-values < 0.05 were considered statistically significant.

## Additional Information

**How to cite this article:** Chen, Q. *et al*. Ultra-high resolution profiles of macular intra-retinal layer thicknesses and associations with visual field defects in primary open angle glaucoma. *Sci. Rep.* **7**, 41100; doi: 10.1038/srep41100 (2017).

**Publisher's note:** Springer Nature remains neutral with regard to jurisdictional claims in published maps and institutional affiliations.

## Figures and Tables

**Table 1:** Participant Demographics.

	POAG Group	Normal Group	P Value
Eyes, n	56	63	—
Number of Subjects (Female/Male)	34 (15/19)	33 (18/15)	0.393
Age, years	36.38 ± 11.55 (19~68)	33.44 ± 10.71 (24~61)	0.295
Spherical Equivalence, D	−1.12 ± 2.12 (−5.50~ + 4.25)	−0.89 ± 1.81 (−5.75~ + 2.50)	0.524
Axial Length, mm	23.80 ± 1.39 (20.84~26.42)	23.84 ± 1.12 (22.18~26.67)	0.845
Visual Field Mean Deviation, dB	−11.00 ± 9.82 (−0.72~−32.16)	NA	—
Best Corrected Visual Acuity (LogMAR)	0.04 ± 0.10 (−0.08~0.40)	−0.01 ± 0.03 (−0.08~0.05)	< 0.001
Intraocular Pressure, mmHg	16.52 ± 4.44 (7.6~26.2)	14.52 ± 1.85 (7.2~20.1)	0.07
Duration, years	3.88 ± 2.91 (0.5~11)	NA	—
**Treatment**			
Drug, n	36	NA	—
Surgery + Drug, n	20		

POAG, primary open angle glaucoma; D, dioptre; NA, not application. Except for the number of subjects, values in parentheses are ranges.

**Table 2:** Repeatability of intra-retinal layer thicknesses measured by automated algorithm in the primary open angle glaucoma (POAG) group.

Intra-retinal layer	M1 (μm)	M2 (μm)	SD of differences (μm)	ICC	COR (%)
**Horizontal Meridian**					
NFL	15.26 ± 3.03	15.92 ± 3.34	0.746	0.986	4.79
GCL + IPL	54.15 ± 12.21	53.84 ± 12.37	1.129	0.998	2.09
INL	31.63 ± 5.47	31.88 ± 5.23	1.067	0.990	3.36
OPL	20.32 ± 1.51	20.07 ± 1.84	1.311	0.820	6.49
HFL + ONL	71.14 ± 5.31	71.37 ± 4.26	1.487	0.976	2.09
MEZ	19.93 ± 2.07	19.60 ± 1.61	1.030	0.916	5.21
OS	34.08 ± 4.89	33.58 ± 4.58	0.534	0.997	1.58
IZ + RPE	34.00 ± 3.01	34.52 ± 3.04	0.906	0.977	2.65
Total	280.51 ± 9.54	280.79 ± 9.81	0.716	0.999	0.26
**Vertical Meridian**					
NFL	21.45 ± 6.89	22.12 ± 7.85	1.766	0.986	8.11
GCL + IPL	51.19 ± 8.82	50.10 ± 7.74	2.181	0.982	4.31
INL	32.74 ± 5.97	34.12 ± 5.13	1.838	0.972	5.50
OPL	21.63 ± 1.97	21.19 ± 1.86	1.636	0.777	7.64
HFL + ONL	63.28 ± 4.64	63.05 ± 4.57	1.615	0.968	2.56
MEZ	20.23 ± 2.23	20.11 ± 1.30	1.180	0.883	5.85
OS	34.71 ± 3.85	34.58 ± 3.93	1.321	0.970	3.81
IZ + RPE	32.53 ± 3.23	32.71 ± 3.20	0.589	0.992	1.81
Total	277.76 ± 10.38	277.99 ± 10.28	0.913	0.998	0.33

M1, mean thickness for the first measurement; M2, mean thickness for the second measurement; SD, standard deviation; ICC, intraclass correlation coefficients of repeatability; COR, coefficients of repeatability; n = 10 eyes.

NFL, nerve fibre layer; GCL + IPL, ganglion cell layer and inner plexiform layer; INL, inner nuclear layer; OPL, outer plexiform layer; HFL + ONL, Henle fibre layer and outer nuclear layer; MEZ, myoid and ellipsoid zone; OS, outer segment of photoreceptors; IZ + RPE, interdigitation zone and retinal pigment epithelium/Bruch complex.
